# Recurrent pain in a child with cerebral palsy: Answers

**DOI:** 10.1007/s00467-021-05156-y

**Published:** 2021-07-29

**Authors:** Andrea Trombetta, Simone Benvenuto, Egidio Barbi

**Affiliations:** 1 University of Trieste, Trieste, Italy; 2 Institute for Maternal and Child Health - IRCCS “Burlo Garofolo”, Trieste, Italy

**Keywords:** Child, Abdominal pain, Cerebral palsy, Urinalysis, Struvite stones

## Answers

**1. What is the most likely cause of this child’s pain?**

The most likely cause of this child’s pain is struvite stones. The patient’s history, laboratory tests, and dental and orthopedic evaluations had already ruled out frequent sources of pain for children with cerebral palsy, such as common infections, constipation, abdominal emergencies, caries, bone fractures, and hip dislocation. Children with cerebral palsy are, in general, more prone to be affected by urinary stones, due to several predisposing factors such as hypercalciuria, bone demineralization, dehydration [1], and topiramate treatment for concomitant epilepsy [2]. Struvite stones, in particular, are a subset of kidney stones, composed of magnesium ammonium phosphate (struvite) and calcium carbonate-apatite, which form as a result of urinary tract infections (UTIs) with urease-producing pathogens. It is known that this type of stone is formed quickly, within a few weeks, in the presence of urease-producing bacteria [3], from genera such as *Proteus*, *Providencia*, *Klebsiella*, or *Staphylococcus*. When the production of ammonia increases and the urine pH is high, the solubility of phosphate decreases and struvite stones can develop.

**2. How should the diagnostic workup be completed?**

Diagnostic work-up should include urinalysis, kidney and bladder urine culture, and ultrasonography, which can detect a densely calcified mass, producing marked posterior acoustic shadowing; indeed, a plain radiograph is also able to identify radiopaque images, appearing as branching calcific densities overlying the kidney outline. Stone culture is recommended to identify urease-producing bacteria and direct antibiotic therapy, since bacteria identified by urine culture do not always match those cultured from the stone [4].

**3. What are the best treatment and follow-up for this patient?**

Given the nature of these stones, treatment should include an initial antibiotic regimen, such as amoxicillin–clavulanate or a cephalosporin (e.g., cefixime), before an eventual removal of residual fragments of the stones. Timing and duration of therapy have not been definitively established: 1–2 weeks of oral or gastrostomy administered antibiotics specific for urine culture are recommended, if available, with the addition of broad-spectrum parenteral preoperative antibiotics [5]. Remarkably, stone formation inhibitors such as citrate are metabolized by bacteria and as a result are ineffective against struvite stones. After treating the episode, imaging and urine cultures should be repeated within 3 months to confirm stone-free status or identify recurrence [6].

## Patient outcome

Stone analysis showed struvite aggregates. A urine culture was performed, testing positive for *Providencia stuartii*, a Gram-negative bacteria. Antibiotic treatment with ceftibuten (9 mg/kg/day in a single daily dose) was started for 2 weeks, with no more pain episodes starting from 3 days after. A kidney ultrasound did not reveal any other endoluminal stones and ruled out a pelvic or ureteral dilatation. No additional episodes were noted in the following year, and follow-up ultrasound scans did not reveal vesicoureteral reflux or incomplete bladder emptying.

## Discussion

The prevalence of struvite stones in children has decreased over the past decades: in France, they accounted for 11% of all urinary stones in the 1980s and then reduced to 6% in the 2000s [7]. In a retrospective analysis, Gnessin et al. [8] showed how immobile patients with musculoskeletal anomalies were prone to form struvite stones (18.4% vs. 6.2% in the control group). This event is due to the several risk factors of UTIs in this population, such as incomplete bladder emptying, vesicoureteral reflux, catheterization, and neurogenic bladder [9]. Clinical presentation of struvite stones substantially differs from other stone types: typical renal colic is not always present, while flank or abdominal pain accounts for nearly 70%, followed by fever (26%) and gross hematuria (18%) [6]. In the case of struvite staghorn calculi, percutaneous nephrolithotomy is considered the gold standard, while extracorporeal shockwave lithotripsy may be useful in selected cases to avoid the surgical approach, especially in pediatric patients given the higher rate of success compared to adults [10].

Routine prevention of kidney stones includes an adequate fluid intake to reduce urine solute concentration and a low sodium diet with limited animal protein and adequate calcium and potassium intake to reduce urinary calcium excretion [11]. Different options have been proposed to reduce the recurrence rate of struvite stones. One is represented by the use of bacterial urease inhibitors such as acetohydroxamic acid, which decreases urinary alkalinity and ammonia levels even in the presence of infection. Unfortunately, its use has been related to serious adverse effects in 20% of cases [5], and the drug should not be used in patients with decreased glomerular filtration rate [12]. A dietary regimen with a low phosphorous and low calcium diet in conjunction with oral estrogens and aluminum hydroxide gel consumption has also been suggested to decrease substrate excretion in urine [13]. However, this cumbersome approach carries adverse effects such as constipation and hypercalciuria. A more feasible option is represented by prophylactic antibiotic therapy because of the relationship between persistent UTI and staghorn stone recurrence [14]. However increasing evidence of struvite kidney stones sustained by bacteria resistant to first- and second-generation cephalosporins suggests that this approach may be of limited effectiveness in the long term [15]. Intermittent self-catheterization is associated with lower rates of UTI than an indwelling urethral catheter in patients with neurogenic bladder requiring catheter-based drainage [16].

Urine analysis to detect an infection and kidney and bladder US to rule out stones should be systematically considered in patients with cognitive impairment with unexplained pain.

Some of this paper has been posted on the ResearchSquare preprint server. The preprint can be accessed here: https://www.researchsquare.com/article/rs-32787/v1.
